# Cost-Effectiveness Analysis of Adding Low Dose Ribavirin to Peginterferon Alfa-2a for Treatment of Chronic Hepatitis C Infected Thalassemia Major Patients in Iran

**DOI:** 10.5812/hepatmon.10236

**Published:** 2013-09-01

**Authors:** Alireza Mehrazmay, Seyed Moayed Alavian, Maziar Moradi-Lakeh, Mahdi Mokhtari Payam, Amir Hashemi-Meshkini, Bita Behnava, Seyyed Mohammad Miri, Pegah Karimi Elizee, Seyed Vahid Tabatabaee, Maryam Keshvari, Kamran Bagheri Lankarani

**Affiliations:** 1 Baqiyatallah Research Center for Gastroenterology and Liver Disease, Baqiyatallah Hospital, Tehran, IR Iran; 2 Department of Community Medicine, School of Medicine, Gastrointestinal and Liver Disease Research Center (GILDRC), Iran University of Medical Science, Tehran, IR Iran; 3 Department of Pharmacoeconomics and Pharmaceutical Administration, Faculty of Pharmacy, Tehran University of Medical Sciences, Tehran, IR Iran; 4 Non-Communicable Disease Research Center, Endocrinology and Metabolism Research Institute, Tehran University of Medical Sciences, Tehran, IR Iran; 5 Health Policy Research Center, Shiraz University of Medical Sciences, Shiraz, IR Iran

**Keywords:** Hepatitis C, PEG-Interferon Alfa-2a, Ribavirin, Cost-Benefit Analysis, Decision Support Techniques, Iran

## Abstract

**Background:**

The prevalence of hepatitis C in Iran is 1% and 18% in general population and thalassemia patients respectively. The cost effectiveness analysis of adding Ribavirin to Peginterferon alfa-2a (PEG IFN alfa-2a) as a combination treatment strategy of chronic hepatitis C in thalassemia patients in comparison with monotherapy could help clinicians and policy makers to provide the best treatment for the patients.

**Objectives:**

In this study we aimed to assess whether adding Ribavirin to PEG IFN alfa-2a is a cost effective strategy in different genotypes and different subgroups of 280 patients with chronic hepatitis C infection from the perspective of society in Iranian setting.

**Patients and Methods:**

A cost effectiveness analysis including all costs and outcomes of treatments for chronic hepatitis C infected thalassemia major patients was conducted. We constructed a decision tree of treatment course in which a hypothetical cohort of 100 patients received “PEG IFN alfa-2a” or “Peg IFN alfa-2a plus Ribavirin.” The cost analysis was based on cost data for 2008 and we used 9300 Iranian Rials (IR Rial) as exchange rate declared by the Iranian Central Bank on that time to calculating costs by US Dollar (USD). To evaluate whether a strategy is cost effective, one time and three times of GDP per capita were used as threshold based on recommendation of the World Health Organization.

**Results:**

The Incremental Cost Effectiveness Ratio (ICER) for combination therapy in genotype-1 and genotypes non-1 subgroups was 2,673 and 19,211 US dollars (USD) per one Sustain Virological Response (SVR), respectively. In low viral load and high viral load subgroups, the ICER was 5,233 and 14,976 USD per SVR, respectively. The calculated ICER for combination therapy in subgroup of patients with previously resistant to monotherapy was 13,006 USD per SVR. Combination therapy in previously resistant patients to combination therapy was a dominant strategy.

**Conclusions:**

Adding low dose of Ribavirin to PEG IFN alfa-2a for treatment of chronic hepatitis C patients with genotype-1 was “highly cost effective” and in patients with low viral load and in previous monotherapy resistant patients was “cost effective.”

## 1. Background

Infection with hepatitis C virus (HCV) could end up with both acute and chronic hepatitis. Although acute infection rarely causes hepatic failure but in 75 – 85 percent of cases it would be developed to chronic infection (1). Chronic HCV infection is usually slowly progressive and in most of patients may not result in clinically apparent liver disease; however, cirrhosis would occur in approximately 20 – 30 percent of chronically infected patients over 20 – 30 years. The prevalence of hepatitis C varies between different regions but the World Health Organization (WHO) has estimated the number of chronically infected hepatitis C patents around 150 million globally (2) which is consistent with other studies (3). Its prevalence in Iran is less than 1% in general population (4). The prevalence of chronic hepatitis C infection in patients with beta thalassemia major is 18%, particularly because of blood transfusion before HCV serological testing (1, 5).

Hepatitis C imposes considerable burden to the global healthcare system in both acute and chronic forms. Global disability adjusted life years (DALYs) per 100,000 population in 2010 in comparison with 1990 has been estimated to have 44.4% growth in acute hepatitis C and also 21.3% and 1.9% growth in liver cancer and cirrhosis of the liver secondary to hepatitis C, respectively (6). This disease also reduces the quality of life in patients (7).

Treatment with antiviral agents could eradicate the virus in the serum and hepatocytes. There are several regimens to treat chronic hepatitis C. Most recently, pegylated interferon (PEG-IFN) as a new generation IFN has shown improvement in response rate (8). Pegylation of IFN also extends the half-life of medicine from a few hours to several days, therefore the injection intervals is decreased from 3 times a week to only once a week (9) which could increase patients' compliance. Peginterferon alfa-2a (PEG IFN alfa-2a) and peginterferon alfa-2b are the two types of PEG-IFN which are both approved by the US Food and Drug Administration (FDA) in treatment of hepatitis C which are different in some structural and pharmacokinetic characteristics (10) as well as effectiveness properties (11-15). Combination (or dual) therapy with PEG-IFN plus Ribavirin as an antiviral medicine has shown a promising effectiveness and also acceptable compliance in many studies (16), and also in Iranian patients (17). In spite of availability and utilization of these medical treatments in Iran, there is not any published economic evaluation to compare these alternatives from Iranian perspective.

## 2. Objectives

In this study, we aimed to analyze the cost effectiveness of treatment with PEG IFN alfa-2a plus Ribavirin in comparison with PEG IFN alfa-2a alone in hepatitis C infected thalassemia patients in Iran.

## 3. Patients and Methods

**Figure 1.**
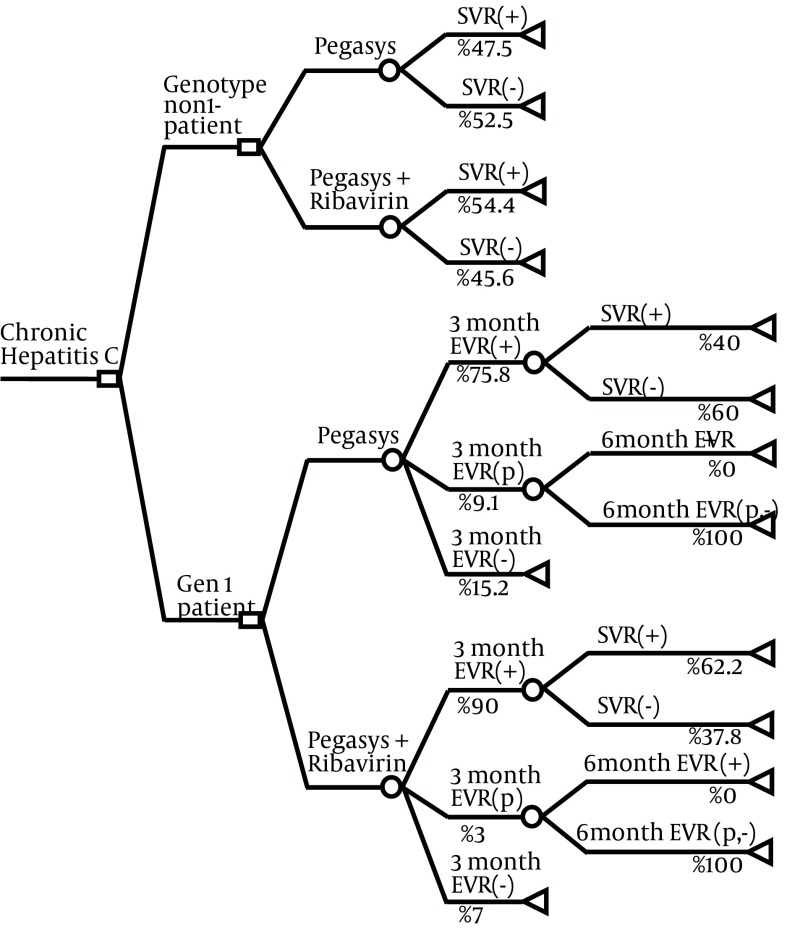
Decision Tree Indicating the Treatment Strategies Included in This Model The square at the root is the decision node; circles depict chance nodes and triangles are terminal nodes.

We developed a decision analytic model to assess the cost and effectiveness of two available treatment strategies which are:

1. Weekly subcutaneous injection of PEG IFN alfa-2a (Pegasys).

2. Weekly subcutaneous injection of PEG IFN alfa-2a plus daily use of Ribavirin oral tablets (see Figure 1). 

The society was used as perspective of study. To develop decision tree model and all analysis, we used Tree Age Pro 2011 and Microsoft Excel 2007.

### 3.1. Patients and Treatment Strategies

This model was designed and run for a hypothetical cohort of 100 thalassemia major patients with diagnosis of chronic hepatitis C infection. Combination treatment strategy was defined as receiving 180 microgram (μg) of PEG IFN alfa-2a, subcutaneously once a week in combination with oral Ribavirin (600 – 800 mg per day based on patients’ hemoglobin level). In this strategy, patient with genotype-1 infection would be treated for 48 weeks and the other genotypes for 24 weeks. Monotherapy strategy was defined as receiving 180 μg of PEG IFN alfa-2a for 48 weeks.

A baseline viral load would be obtained from patients before beginning the treatment. In genotype-1 patients, the viral load test would be repeated after three months of therapy and compared with the baseline. In patients with complete Early Virological Response (EVR), indicating no detectable HCV RNA, or partial EVR, indicating at least a two log reduction from baseline viral load, the treatment would be continued because there is a high probability of success at the end of treatment course. In other patients who do not achieve complete or partial EVR, treatment success is highly unlikely, therefore treatment would be stopped because of potential adverse effects and costs and very low probability of success at the end. For partial responder patients, this procedure would be repeated again in sixth month and for patients without complete EVR at sixth month, treatment would be discontinued. Checking EVR would be less useful in genotypes non-1 due to the shorter treatment course and higher response rates. So treatment of genotype-1 patients would be discontinued at third month for patients in the category of no response, and at sixth month for partial responders who do not achieve complete response. More details about the treatment strategies are represented elsewhere (18).

### 3.2. Data Sources for Decision Tree Model

#### 3.2.1. Effectiveness and Outcomes of Interest

All effectiveness and safety data including different probabilities were extracted from a domestic clinical trial, conducted in Baqiyatallah University of Medical Sciences in 2007 which was about combination treatment of HCV in 180 thalassemia major patients (18). We did not use any international evidence, mostly because of less similarity with real situation of Iranian setting of treatment. The time period of this model was until the end of follow up course, which was 72 weeks in most of the patients so the longer time side effects were not taken into account. In this study the patients were categorized regarding three parameters: HCV genotype (genotype-1 and non-1), previous treatment outcome (naïve, resistant to previous monotherapy and resistant to combination therapy), and the basic viral load (low viral load (< 600,000 IU/mL) and high viral load (> 600,000IU/mL).

Final outcome was divided into sustain responder (SVR+) and sustain nonresponder (SVR-). Sustain Virological Response (SVR) is defined as no detectable viral level (the absence of hepatitis C RNA) after six months from completion of therapy (success) (19), and Sustain nonresponder (failure) consist of five situations including response then relapse (detected HCV RNA after treatment course), withdrawal (stopped treatment due to adverse effects or no response), no response (positive HCV RNA in all duration of treatment course), breakthrough (relapsed before the end of treatment course), death (death during treatment course).

As proportion of genotype-1 in naïve subgroup (no previous treatment) was not similar to Iranian population, and both cost and effectiveness outcomes are strongly dependent to genotype distribution of patients, comparing these two strategies in this subgroup was not relevant and valuable, therefore we did not calculate and report cost effectiveness ratios for this subgroup. Patients with genotype-1 virus, who consist about 66% of these patients population in Iran, have a much lower probability of SVR than those patients with non-genotype-1 virus (20).

#### 3.2.2. Cost

In this model, only direct medical costs including medication (pharmaceutical) costs, laboratory tests, costs of side effects treatment and physician visits were taken into account and direct non-medical costs, indirect costs and intangible costs were not included in our analysis. Costs of physician visit charges and laboratory tests were extracted from the annual report of Medical Council of Islamic Republic of Iran. Costs of medicines were extracted from the Ministry of Health annual report on pharmaceutical market entitled “Amarnameh.” To include side effect costs into our analysis, we only took physician visits and major medical and laboratory tests into account and the pharmaceutical cost were ignored because their costs were not considerable. The cost analysis was based on cost data for 2008. We also used 9300 Iranian Rials (IR Rial) as exchange rate declared by the Iranian central bank on that time to calculate costs by US Dollar (USD). No discount rate was considered in the cost analysis of this study.

Considering the genotype distribution of different subgroups in the patients, the weighted mean cost for each subgroup and also weighted mean cost of 100 patients were calculated.

### 3.3. Cost Effectiveness Ratios

Two cost effectiveness ratios were calculated based on our decision tree: cost effectiveness ratio (CER) which means cost per one SVR and indicates superiority of one strategy to the other one for cost effectiveness; and incremental cost effectiveness ratio (ICER) which means cost per one more SVR. To evaluate whether one strategy with more cost and more effectiveness or with less cost and less effectiveness is cost effective, we compared the ICER with recommended threshold of the World Health Organization (WHO) for countries without any calculated threshold. In this method, ICER has to be compared with one and three times of GDP per capita and if it was less than one time of GDP per capita, it would be “highly cost effective” and if it was between one time and three times of GDP per capita it would be “cost effective”, but if it was more than three times of GDP per capita, it is “non-cost effective” (21). 4,678 USD was used as GDP per capita of Iran in 2008 based on the World Bank reports. This recommendation of WHO about threshold is mostly for cost utility analysis with “cost per QALY gained” or “cost per DALY averted” as outcome but as there is not any threshold calculated or accepted for Iran, we used it as our threshold.

### 3.4. Sensitivity Analysis

A deterministic one-way sensitivity analysis was conducted to evaluate the sensitivity of the result to the changes in key input variables of the model. The considered key variables in this study were SVR and Positive Predictive Value (PPV) of EVR for combination therapy. PPV of EVR is defined as the proportion of EVR patients (complete or partial) with treatment success at the end of study. PPV could be associated with several internal and external parameters including sensitivity and specificity of laboratory tests, pretest probability, quality of medicines, HCV genotype and patients characteristics. By considering the minimum domain of 95% confidence interval of SVR and PPV, the ICER and also CER would be calculated to see how much the results of our model is dependent to variability of key input parameters regarding their uncertainties.

## 4. Results

SVR and PPV were calculated for two treatment strategies which are showed in the Table 1. As seen in this table, PPV is clearly higher in combination therapy strategy for all subgroups. This means that higher percent of monotherapy group patients, would not achieve SVR and higher cost for each SVR would be imposed to healthcare system and patients. 

**Table 1.** The Comparison Between Combination and Monotherapy for EVR, PPV and SVR in Different Subgroups

Subgroup	Strategy	EVR^a^	SVR^a^ (95% CI^a^)	PPV^a^ (95% CI)
**Genotype-1**	PEG IFN alfa-2a	84.85	29.27% (15.34 – 43.20%)	39.29 (21.20-57.38)
	PEG IFN alfa-2a + RBV^a^	93.00	48.74% (39.72 – 57.76%)	59.14 (49.15-69.13)
**Genotype non-1**	PEG IFN alfa-2a	90.91	47.50% (32.02 – 62.98%)	53.33 (35.48-71.19)
	PEG IFN alfa-2a + RBV	96.97	54.43% (43.45 – 65.41)	59.38 (47.34-71.41)
**Low Viral Load**	PEG IFN alfa-2a	84.21	39.13 (25.03 – 53.23)	40.63 (23.61 – 57.64)
	PEG IFN alfa-2a + RBV	96.88	54.87 (45.69 – 64.04)	60.22 (50.27 – 70.16)
**High Viral Load**	PEG IFN alfa-2a	92.59	38.71 (21.56 – 55.86)	52.00 (32.42 – 71.58)
	PEG IFN alfa-2a + RBV	91.18	44.71 (34.14 – 55.28)	59.68 (47.47 – 71.89)
**Naïve**	PEG IFN alfa-2a	91.43	41.86 (27.11 – 56.61)	50 ( 28.61 – 71.39)
	PEG IFN alfa-2a + RBV	95.92	49.18 (36.63 – 61.73)	57.45 (36.30 – 78.59)
**Resistant to Previous Monotherapy **	PEG IFN alfa-2a	85.71	41.67 (21.94 – 61.39)	55.56 (34.30 – 76.81)
	PEG IFN alfa-2a + RBV	96.36	59.68 (47.47 – 71.89)	69.81 (57.45 – 82.17)
**Resistant to Previous Combination Therapy**	PEG IFN alfa-2a	57.14	21.43 (0 – 42.92)	12.5 (3.6 – 21.4)
	PEG IFN alfa-2a + RBV	91.94	44.59 (33.27 – 55.9)	54.39 (40.98 – 67.80)

^a^ CI, Confidence Interval; EVR, Early Virological Response; PPV, Positive Predictive Value; RBV, Ribavirin; SVR, Sustain Virological Response

Table 2 indicates the cost of different components of HCV treatment in thalassemia major patients in Iran. As it is shown, there is not any considerable difference in medication (pharmaceutical) costs between these two treatment strategies in Iran because Ribavirin is far less expensive than PEG IFN alfa-2a. 

**Table 2.** The Cost Components of HCV Infection Treatment in Thalassemia Major Which Are Included in the Analysis

Service	Detail	Cost (USD^a^)
**Medication Cost**		
Pegasys Injection	Pegasys dose was 180 μg/week	290
Ribavirin Tablet 200 mg	Ribavirin dose for Hb < 10 and Hb > 10 was 600 mg/day and 800 mg/day, respectively	0.34
**Visits And Hospitalization**		
Routine Visits	Gastroenterologist (totally 16 times in treatment duration)	7.4
Consult Visits	Cardiologist, psychiatrist, ophthalmologist and etc. (for adverse effects)	7.4 (9.1 for psychiatrist)
Hospitalization	Due to sepsis, DKA, heart failure, severe depression, hepatic decompensation	Mean cost: 215
**Laboratory Tests**		
Enrollment Tests	Include CBC, TFT, LFT, biochemistry, hepatitis serology, liver biopsy, PCR, lipid profile, LDH, AFP and genotyping)	325.75
Routine Safety Evaluation Tests	CBC, LFT, Ferritin, TG, Cholesterol and FBS	8.3
Occasionally Tests	HCV viral load in third month and PCR in sixth and 12th months of treatment course and third and sixth months of follow up course	139.7
	Thyroid function test (TFT) each 3 months	15
CBC	It was performed weekly until the third month, then 2 times per month until 12th month and monthly in follow up course for combination therapy group.	0.6
**Cost of Supportive Intervention for Adverse Effects**		
Packed Cell	Monotherapy and combination therapy patients' need to packed cell was averagely 33 and 54 units respectively.	129

^a^ Abbreviations: USD, US Dollar

The outcomes of cost effectiveness analysis were calculated for combination and monotherapy strategies in the time period of 72 weeks and are summarized in Table 3. 

**Table 3.** Costs, Effectiveness and Cost Effectiveness Indicators in Subgroups for Two Monotherapy and Combination Therapy Strategies^a ^

Subgroup	Strategy	SVR^b^(95%CI)	Cost for 100 Patients (USD)	CER^b^(USD^b^per one SVR)	ICER^b^(USD per one more SVR)
**Genotype-1**	PEG IFN alfa-2a	29.27% (15.34 – 43.20%)	1,754,180	59,934	2,673
	PEG IFN alfa-2a + RBV^b^	48.74% (39.72 – 57.76%)	1,806,231	37,058	
**Genotype non-1**	PEG IFN alfa-2a	47.50 (32.02 – 62.98)	1,171,742	24,668	19,210
	PEG IFN alfa-2a + RBV	54.43% (43.45 – 65.41)	1,304,872	23,973	
**Low viral load**	PEG IFN alfa-2a	39.13 (25.03 – 53.23)	1,536,399	39,263	5,233
	PEG IFN alfa-2a + RBV	54.87 (45.69 – 64.04)	1,618,766	29,503	
**High viral load**	PEG IFN alfa-2a	38.71 (21.56 – 55.86)	1,482,614	38,300	14,975
	PEG IFN alfa-2a + RBV	44.71 (34.14 – 55.28)	1,572,469	35,173	
**Naïve^c^**	PEG IFN alfa-2a	41.86 (27.11 – 56.61)	1,733,217	NA	NA^b^
	PEG IFN alfa-2a + RBV	49.18 (36.63 – 61.73)	1,690,536	NA	
**Resistant to Previous Monotherapy **	PEG IFN alfa-2a	41.67 (21.94 – 61.39)	1,683,906	40,413	13,005
	PEG IFN alfa-2a + RBV	59.68 (47.47 – 71.89)	1,918,141	32,141	
**Resistant To Previous Combination Therapy^d^**	PEG IFN alfa-2a	21.43 (-0.07 – 42.92)	1,939,034	NA	NA
	PEG IFN alfa-2a + RBV	44.59 (33.27 – 55.92)	1,807,838	NA	

^a^ GDP per capita: 4,678 USD; 3 times of GDP per capita:14,034 USD

^b^ Abbreviations: CER, Cost Effectiveness Ratio; ICER, Incremental Cost Effectiveness Ratio; NA, Not Applicable; RBV, Ribavirin; SVR, Sustain Virological Response; USD: US Dollar.

^c^ In this subgroup, the proportion of genotype-1 was not similar to Iranian population so we did not report any ratio for them

^d^ In this subgroup, combination therapy was a dominant strategy (higher effectiveness with lower cost) so calculation of cost effectiveness ratios was not reported.

Patients with genotype-1: the CER for monotherapy and combination therapy was 59,934 and 37,058 USD per SVR, respectively. So combination therapy with PEG IFN alfa-2a and Ribavirin needed less cost to achieve SVR. The calculated ICER for combination therapy was 2,673 USD per SVR that is less than GDP per capita. It means that, adding Ribavirin to PEG IFN alfa-2a could be considered as “highly cost effective” strategy in genotype-1 patients.

Patients with genotype non-1: CER for monotherapy and combination therapy was 24,668 and 23,973 USD per SVR, respectively. So combination therapy with PEG IFN alfa-2a and Ribavirin needed less cost to achieve SVR. But ICER for combination therapy was 19,210 USD per SVR which was higher than three times of GDP per capita implying that adding Ribavirin to PEG IFN alfa-2a does not seem to be a “cost effective” strategy for these genotypes in Iran.

Low basic viral load: CER for monotherapy and combination therapy was 39,263 and 29,503 USD per SVR, respectively. So combination therapy with PEG IFN alfa-2a and Ribavirin needed less cost to achieve SVR. The ICER for combination therapy was 5,233 USD per SVR that was lower than three times per capita GDP. So adding Ribavirin to PEG IFN alfa-2a could be “cost effective.”

High basic viral load: CER for monotherapy and combination therapy was 38,300 and 35,173 USD per SVR, respectively. So combination therapy with PEG IFN alfa-2a and Ribavirin needed less cost to achieve SVR. But ICER for combination therapy was 14,975 USD per SVR which was higher than three times of GDP per capita so adding Ribavirin to PEG IFN alfa-2a could not be considered as a “cost effective strategy.”

Naïve patients: In this subgroup, the proportion of genotype-1 patients in the sample was not similar to society so we could not compare two strategies because of different duration of treatment and useless result for policy makers in Iran.

Resistant to previous monotherapy: CER for monotherapy and combination therapy was 40,413 and 32,141 USD per SVR, respectively. So combination therapy with PEG IFN alfa-2a and Ribavirin needed less cost to achieve SVR. The ICER for combination therapy was 13,005 USD per SVR which was lower than three times per capita GDP so adding Ribavirin to PEG IFN alfa-2a in this category of patients is a “cost effective” strategy in Iran.

Resistant to previous combination therapy: In this subgroup, combination therapy was a dominant strategy meaning that it had more effectiveness and less cost compared to monotherapy which did not need any economic evaluation.

### 4.1. Sensitivity Analysis

For sensitivity analysis, we calculated the ICER with the minimum amount of SVR and PPV in their 95% confidence interval. In minimum amount of PPV, combination therapy in genotype non-1 would be dominated and ICER in all other groups would be higher than three times of GDP per capita. In minimum amount of SVR in 95% confidence interval, combination therapy in genotype non-1 and high viral load subgroups would be dominated and in all other subgroups ICER would be higher than three times of GDP per capita. In Table 4, the calculated CER and ICER with minimum amount of SVR and PPV in several subgroups are shown. 

**Table 4.** Sensitivity Analysis: Calculated CER and ICER by Minimum Domain of 95% Confidence Interval of PPV and SVR^a ^

Subgroup	Monotherapy	Combination Therapy			
	CER^b^ (USD^b^per SVR)	Minimum PPV^b^in 95%CI		Minimum SVR^b^in 95%CI	
		CER (USD per SVR)	ICER^b^(USD per one more SVR)	CER (USD per SVR)	ICER (USD per one more SVR)
**Genotype-1**	59,934	39,523	316,773	45,473	497,982
**Genotype non-1**	24,668	28,428	Dominated	30,033	Dominated
**Low Viral Load**	39,263	33,239	860,725	35,427	1,255,309
**High Viral Load**	38,300	36,332	1,966,050	46,064	Dominated
**Resistant to Previous Monotherapy **	40,413	34,654	1,711,831	40,410	4,038,486

^a^ GDP per capita: 4,678 USD; 3 times of GDP per capita:14,034 USD.

^b^ Abbreviations: CER, Cost Effectiveness Ratio; ICER, Incremental Cost Effectiveness Ratio; PPV, Positive Predictive Value; SVR, Sustain Virological Response; USD, US Dollar.

## 5. Discussion

This study indicated that although in all subgroups, combination therapy was more effective than monotherapy, but combination therapy of HCV in thalassemia major patients could be considered as a “highly cost effective” strategy only in genotype-1 patients and as a “cost effective” strategy in low basic viral load patients and also in patients with previous resistance to monotherapy. It was shown that in patients with previous resistance to combination therapy, it is a dominant strategy to put them therapy again in comparison with monotherapy.

The other studies comparing monotherapy and combination therapy regimens in hepatitis C patients have shown that combination therapy is the most cost effective treatment in the patients with genotypes 2, and 3 and low viral load patients (21-24); however, our study indicated that in HCV infected thalassemia major patients it was for the patients with genotype-1 and low viral load patients and previous monotherapy resistant patients. One limitation of our study was that the proportion of genotype-1 was not similar to HCV infected thalassemia major population of Iran in naïve subgroup; therefore we were not able to generalize the result of this study to Iranian setting whether combination therapy is cost effective in this subgroup. As this is an applied study to be used only by policy makers in Iran, we decided not to calculate and report the ICER for this subgroup.

This analysis was based on the clinical trial evaluating both monotherapy and combination therapy group with the same inclusion criteria. As the types and severity of adverse events experienced by patients who were exposed to PEG IFN alfa-2a and Ribavirin might be varied, we tried to include consideration of adverse events which could be resulted from treatment in the analysis. Using clinical data from a domestic clinical trial could be considered as an advantage for this study because of more similarity with real setting of Iran, but has its own weaknesses. One limitation was that only one domestic clinical trial was found which was not multicenter, however we tried to overcome this limitation by sensitivity analysis. The number of patients in this clinical trial was also another limitation especially when we categorized them into subgroups which caused relatively wide 95% confidence interval. Another limitation of this study was that we did not take patients' quality of life into consideration. In one study evaluating the effects of PEG IFN alfa-2a plus Ribavirin in patients with chronic hepatitis C on their quality of life, the results indicated that quality of life of these patients was significantly improved (25). Using more comprehensive outcome of interest (i.e. quality adjusted life years (QALY) or disability adjusted life years (DALY)) could provide better understanding about different aspects of disease and treatment effects. Also using more complicated decision support models including Markov models which consider more details of disease could result in more accurate output for policy makers.

This study was based on cost data of 2008, so considering much market variability in the health sector of Iran in the last years, the results may not be easy to defend in 2013. However it is a challenge over economic evaluation studies in the countries with rapidly changing or unstable economic situation.

Although both medicines are available in Iran, this study is the first economic evaluation of two common treatment strategies for HCV infection in thalassemia patients. The results of this analysis could be used by policy makers in setting reimbursement strategies and guideline development. More analysis is suggested to be conducted by considering more domestic and also international evidences, using more complicated models and more comprehensive outcomes and perspective for more accurate results. This study indicated that adding low dose of Ribavirin to PEG IFN alfa-2a for treatment of chronic hepatitis C infection in thalassemia is highly cost effective in genotype-1 patients and also cost effective in patients with low viral load or with previous monotherapy resistance. But in patients with genotype non-1 or high viral load, adding Ribavirin to PEG IFN alfa-2a could not be considered as a cost effective strategy.
